# Inflammatory Myositis Following Statin Use in a Patient With Untreated Hypothyroidism

**DOI:** 10.7759/cureus.48463

**Published:** 2023-11-07

**Authors:** Mellisa Renteria, Misbah Jilani, Michael J Brockman, Harry E Davis

**Affiliations:** 1 Internal Medicine, Texas Tech University Health Sciences Center Paul L. Foster School of Medicine, El Paso, USA

**Keywords:** atypical presentation of hypothyroidism, statin-associated muscle symptoms, statin-associated autoimmune myopathy, statin-associated necrotizing autoimmune myositis, drug-induced myositis, hypothyroid patients, statin-induced myopathy, drug-induced myopathy, statin induced rhabdomyolysis, inflammatory myositis

## Abstract

Inflammatory myositis (IM) presents a diagnostic challenge due to its multifaceted etiology and varying clinical presentations. This case involves a 55-year-old male with asymptomatic hypothyroidism, recent statin use, and rapidly progressing proximal muscle weakness. He presented with profound weakness in the upper and lower extremities, severely impairing his daily activities. The patient's medical history included recent hospitalizations for idiopathic interstitial lung disease, myopericarditis, and pneumonia, adding complexity to his condition. Laboratory findings revealed elevated serum muscle enzymes, notably creatine kinase, indicating muscle damage and rhabdomyolysis. Serological testing confirmed the absence of myositis-specific antibodies and anti-3-hydroxy-3-methylglutaryl-coenzyme A reductase (HMGCR) antibodies. The patient was eventually diagnosed with IM and rhabdomyolysis, likely secondary to statin use or hypothyroidism. Treatment with methylprednisolone, levothyroxine, and discontinuation of atorvastatin led to rapid improvements in AST levels and overall muscle strength. This case highlights the challenges of managing IM and emphasizes the importance of assessing thyroid function before initiating lipid-lowering therapy.

## Introduction

Inflammatory myositis (IM) encompasses a diverse group of autoimmune disorders with varying clinical presentations, treatment responses, and prognoses [1,2]. Muscle weakness is the primary clinical hallmark, but IM can also affect multiple organs, including the skin, joints, lungs, heart, and gastrointestinal tract [1,3]. IM often co-occurs with various conditions and has been linked to statin therapy [4].

Hypothyroidism is a known contributor to secondary dyslipidemia, which is associated with atherosclerosis and can result in statin-induced myopathy (SIM) and spontaneous myopathy [3,4]. Furthermore, factors such as concurrent use of fibrates, inhibitors of hepatic cytochrome P-450 (CYP450), major trauma, and surgery further increase the risk of SIM [4]. Previous research indicates that the prevalence of SIM in the general population falls within the range of 0.1% to 0.2%, while the incidence of hypothyroid-induced myopathy remains uncertain [3-5]. Given these associations, it is essential to consider patients' thyroid status before initiating lipid-lowering medications. For individuals already on statin therapy, vigilant monitoring of thyroid function is crucial when myopathic symptoms or an inadequate response to lipid-lowering treatment become evident.

## Case presentation

A 55-year-old male, previously in good health until two months before admission, presented to the emergency department with a rapidly evolving clinical course. He described an overwhelming weakness affecting both his upper and lower extremities, leaving him unable to rise from a seated position, perform basic shoulder and elbow movements, or even manage simple tasks like combing his hair. Notably, his decline had begun two months earlier when he experienced a ground-level fall, necessitating the use of a cane for mobility.

**Figure 1:**
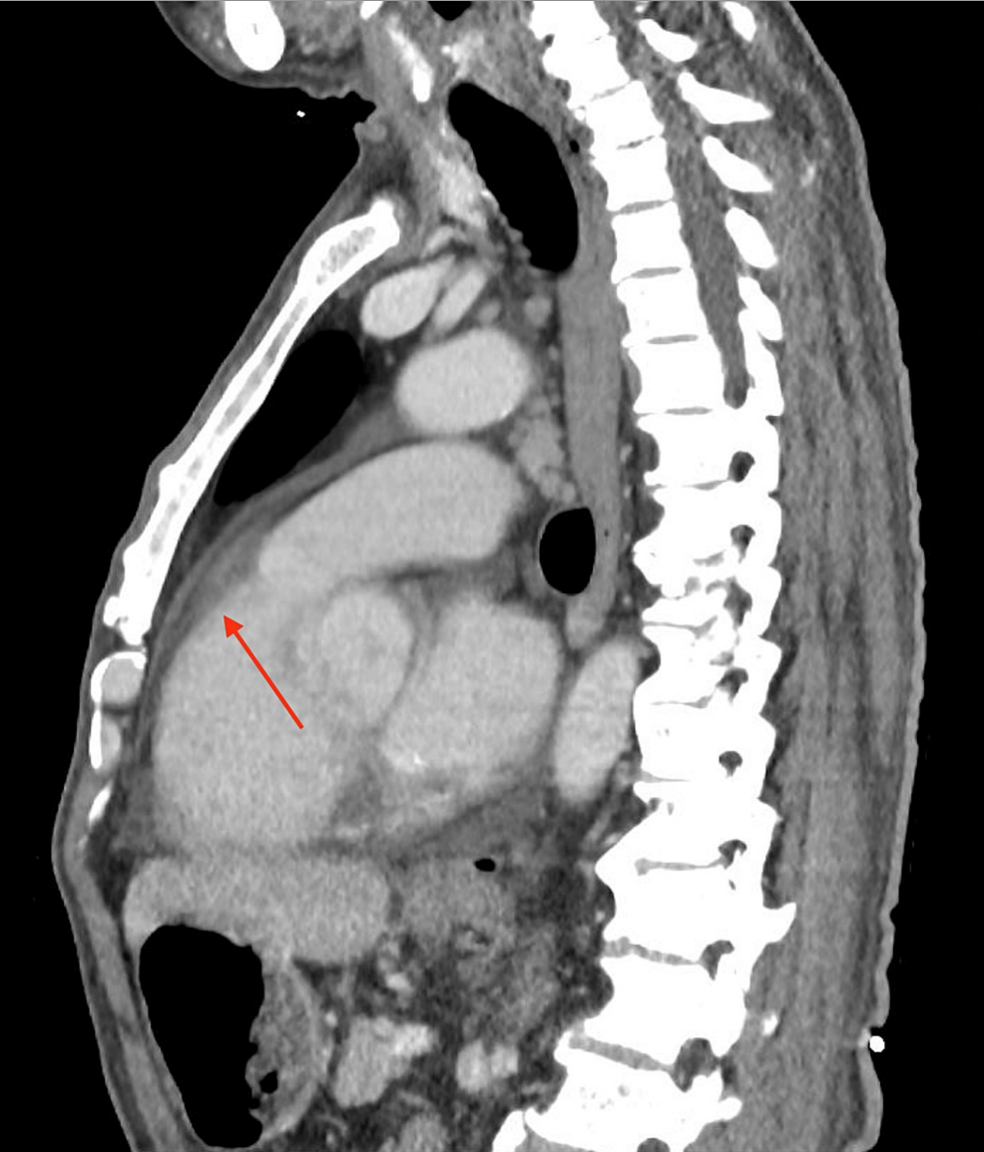
CT thorax displaying pericardial effusion (red arrow). CT: computed tomography.

**Figure 2:**
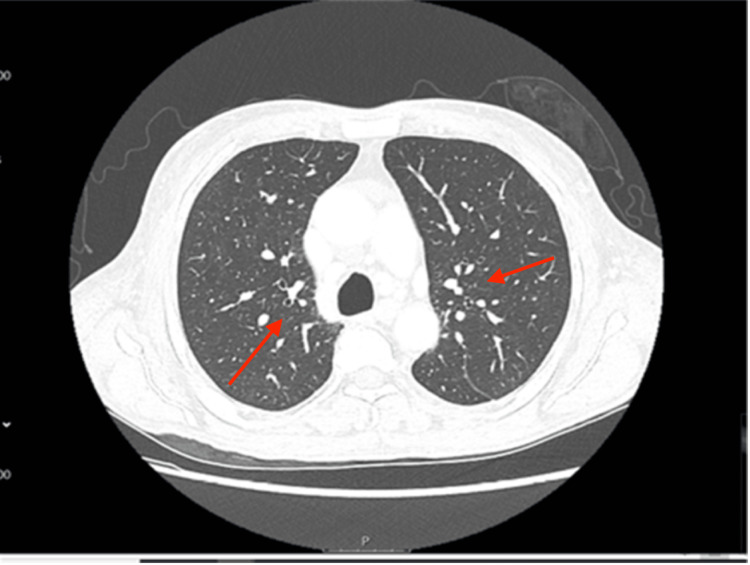
CT thorax with contrast showing interstitial pneumonia pattern of interstitial lung disease (red arrow). The image revealed no pulmonary nodules, masses, or focal airspace consolidations. However, the underlying pulmonary infectious process could not be excluded. In addition, the image revealed cardiomegaly with pericardial effusion and mediastinal lymphadenopathy. CT: computed tomography.

Remarkably, this patient had recently undergone two prior hospitalizations. Approximately two months before his current presentation, he received a diagnosis of idiopathic interstitial lung disease and myopericarditis, a confirmation reached through a contrast-enhanced CT thorax (Figures 1, 2). The patient had endured an episode of myopericarditis following a left heart catheterization for elevated troponin levels, which confirmed normal coronary arteries. Consequently, he had been placed on a regimen comprising colchicine, atorvastatin, and metoprolol. The inclusion of short-term colchicine, alongside a statin and beta-blocker, followed established medical therapy shown to confer benefits in preventing heart disease, particularly among individuals with interstitial lung disease and concurrent viral infections. Just four days before his current presentation, the patient was diagnosed with pneumonia and initiated on a doxycycline antibiotic regimen.

Upon admission, the patient's vital signs remained unremarkable, with oxygen saturation on room air at 97%. Physical examination unveiled proximal muscle weakness, with specific assessments highlighting shoulder abduction and adduction at 3/5, elbow flexion at 4/5, elbow extension at 3/5, and hip flexion, abduction, adduction, extension, and flexion all at 4/5. Furthermore, the patient exhibited weakness in neck flexion and extension and the inability to rise from a seated position with arms folded, indicative of significant motor impairment.

Table 1 displays significant laboratory values, pointing to an inflammatory process upon admission. Notably, laboratory evaluation uncovered elevated serum muscle enzymes, including creatine kinase (CK) levels, which indicated muscle damage and rhabdomyolysis. A thorough viral workup investigating hepatitis and respiratory infections yielded unremarkable results. Echocardiogram results revealed no anomalies, with an ejection fraction of 65%-70%. An autoimmune panel aimed at uncovering underlying autoimmune myositis yielded negative results for the anti-Jo-1 antibody, SSA (Ro), SSB (La), CCP, ANCA, RF, ANA, and anti-HMGCR antibody IgG.

**Table 1:** Significant laboratory values on admission.

Tests	Results	Normal Range
Creatine kinase	3200 U/L	55 - 170 U/L
Erythrocyte sedimentation rate (ESR)	104 mm/hour	0 - 19 mm/hour
Ferritin	2490 ng/mL	17.9 - 464 ng/mL
C-reactive protein	3.14 mg/dL	0 - 1 mg/dL
TSH	121 mIU/L	0.465 - 4.68 mU/L
Free T4	0.32 ng/dL	0.78 - 2.19 ng/dL
Aspartate aminotransferase (AST)	89 IU/L	17 - 59 IU/L
Alanine aminotransferase (ALT)	307 IU/L	0 - 50 IU/L
Alkaline phosphatase	140 IU/L	38 - 126 IU/L
C3	49 mg/dL	88 - 165 mg/dL

**Figure 3:**
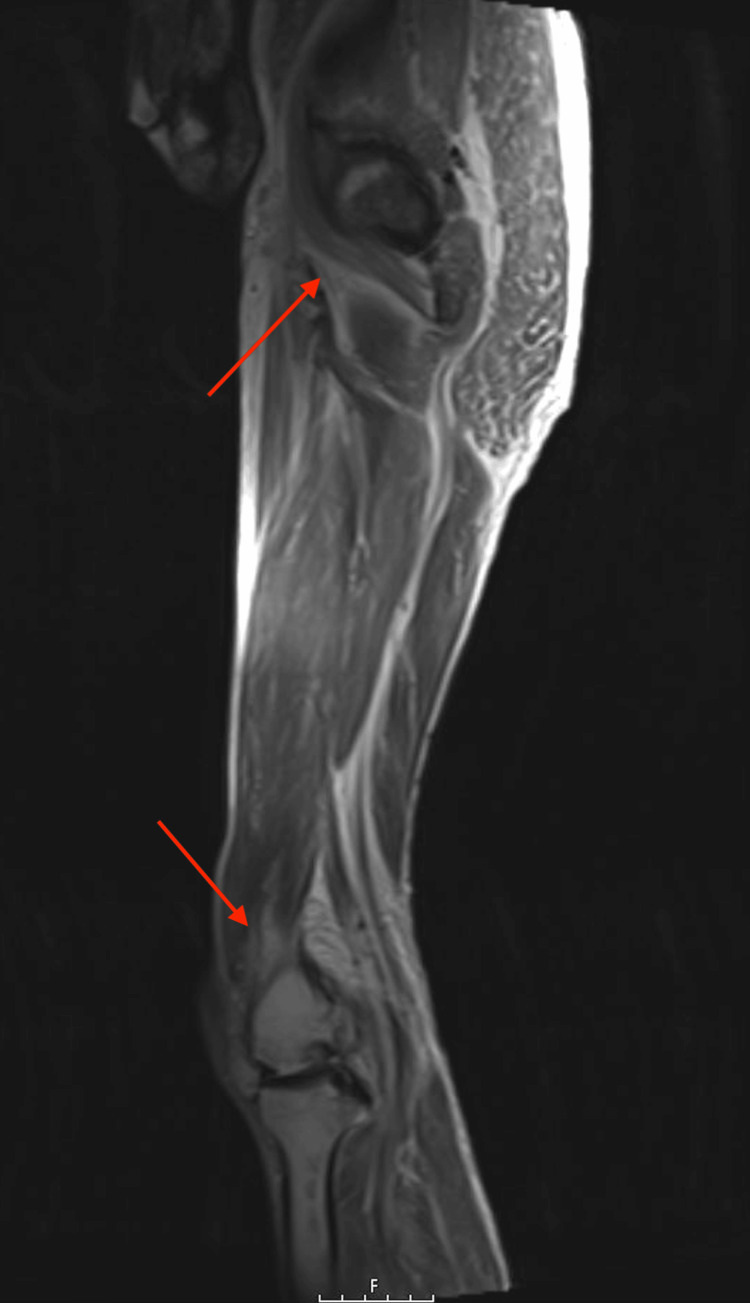
MRI left lower extremity non-joint without contrast showing decreased T1 and increased T2 signal within the subcutaneous adipose tissue (red arrow). MRI: magnetic resonance imaging.

**Figure 4:**
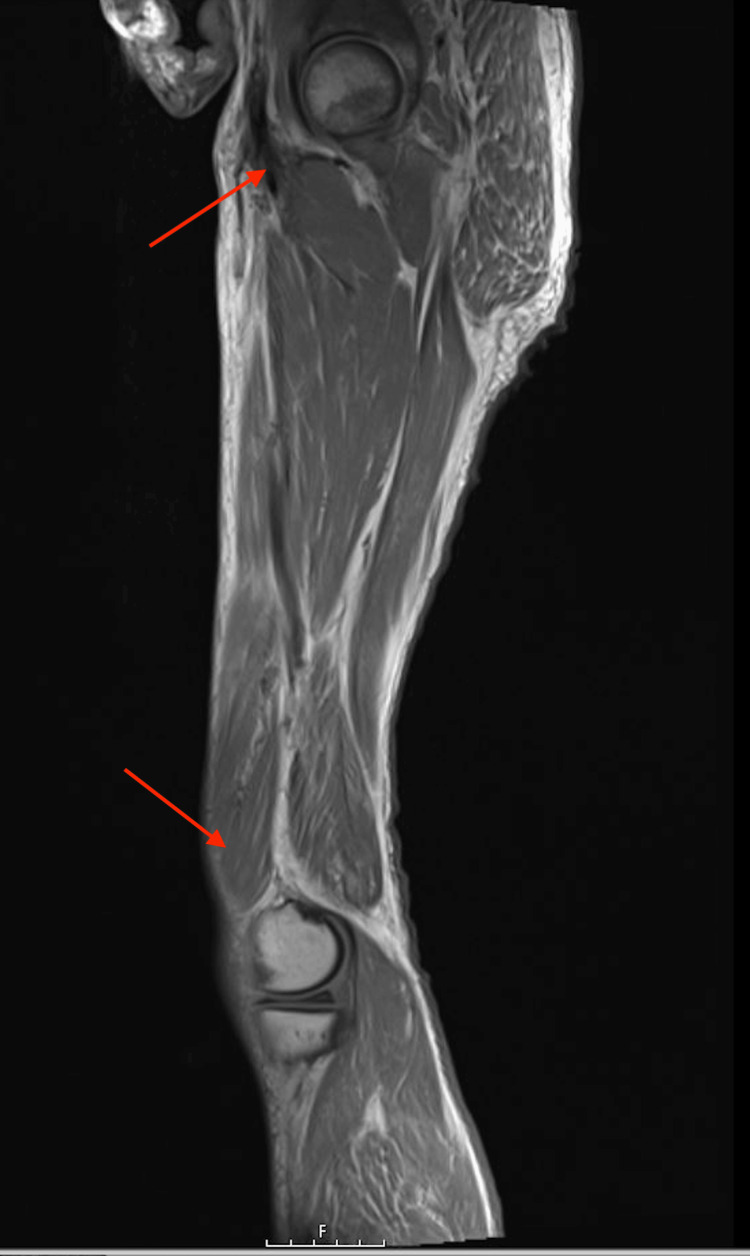
MRI right lower extremity non-joint without contrast should diffuse a decreased T1 and increased T2 signal within the subcutaneous adipose tissue (red arrow). MRI: magnetic resonance imaging.

Magnetic resonance imaging (MRI) of the left lower extremity (Figure 3) and right lower extremity (Figure 4) displayed a diffuse decreased T1 and increased T2 signal within the subcutaneous adipose tissue lateral to the left hip and proximal femur. This signal extended into the deep fascial planes and thigh musculature, consistent with myositis and necrotizing fasciitis. Subsequently, a percutaneous ultrasound-guided right anterior thigh muscle biopsy was conducted, confirming the presence of fragments of skeletal muscle fibers and adipose tissue, along with a few foci of chronic inflammation. These diagnostic findings underscore the complexity of arriving at a definitive diagnosis in cases of inflammatory myositis and rhabdomyolysis, particularly when multiple potential etiologies are at play.

Given the patient's proximal muscle weakness, elevated serum muscle enzymes, and the muscle biopsy findings, autoimmune necrotizing myositis (ANM) was initially suspected. However, the presence of rhabdomyolysis, elevated AST, and the rapid improvement of AST levels from the 600s to the 400s, coupled with the emergence of new-onset hypothyroidism and the use of myotoxic drugs (specifically, statin and colchicine), rendered ANM less likely. This led to a diagnosis of inflammatory myositis and rhabdomyolysis, with the etiology likely stemming from statin use or hypothyroidism.

The patient's treatment plan was initiated with methylprednisolone at a dosage of 500 mg twice daily for a period of three days. Additionally, levothyroxine was administered at 50 mcg. The patient's pre-admission medications, which included colchicine at a dosage of 0.5 mg twice daily and metoprolol at 25 mg twice daily, were resumed as part of the treatment regimen, while atorvastatin was discontinued.

Upon the successful completion of the three-day course of prednisone, the patient experienced a notable improvement in weakness. Subsequently, the patient was discharged with a prescription for prednisone at 60 mg and levothyroxine at 50 mcg, with scheduled outpatient follow-up in four weeks. During the follow-up appointment, the patient reported a significant enhancement in strength and no longer required the use of a cane.

## Discussion

IM constitutes a range of autoimmune disorders affecting skeletal muscles, marked by inflammation, muscle weakness, and atrophy [3]. Despite advancements in diagnostic techniques, achieving a definitive diagnosis remains challenging due to the presence of multiple potential causes and overlapping symptoms [3]. IM may manifest in conjunction with connective tissue diseases such as systemic lupus erythematosus and Sjogren's syndrome, as well as in association with malignancies, drug toxicity, and infectious agents [4-6]. The intricate etiology of this condition has been extensively examined in scientific literature, highlighting the intricacy and difficulties involved in arriving at a conclusive diagnosis [1-3].

The utilization of statins as a therapeutic intervention has emerged as a prominent cause of muscle-related adverse events in individuals with myositis [7]. Hyperlipidemia is a major risk factor for cardiovascular morbidity and mortality, and although statins are the first-choice therapy for dyslipidemias and considered the cornerstone of atherosclerotic cardiovascular disease, patient compliance is often poor due to SIM [7,8]. Although statins are generally considered safe and well-tolerated, neuromuscular side effects represent about two-thirds of all adverse events [8]. These side effects include cramps, myalgia, weakness, immune-mediated necrotizing myopathy, and, more rarely, rhabdomyolysis [7]. In addition, statins may lead to peripheral neuropathy and induce or unmask pre-existing neuromuscular junction dysfunction [9]. Hence, clinical follow-up of patients taking statins is essential to detecting early side effects that may cause neuromuscular damage [8,9].

With the increasing use of statins in clinical settings, it is expected that the incidence of neuromuscular complications will increase as well [8]. Therefore, research is underway to develop pharmacogenomic and environmental studies to predict neuromuscular complications due to statin exposure in a timely manner, leading to a more personalized therapeutic approach [9]. Nonetheless, de-challenge or cessation of statins or alternative use of other lipid-lowering agents can help avoid adverse events in patients who experience muscle-related side effects from statins. It raises the question of whether initiation of statin therapy in this individual caused or exacerbated the final diagnosis [4]. 

Hypothyroidism is a well-established cause of secondary dyslipidemia with a recognized link to atherosclerosis [4,10]. Patients with hypothyroidism often exhibit elevated levels of apolipoprotein B100-containing lipoproteins, including very low-density lipoprotein and low-density lipoprotein. Additionally, hypothyroidism serves as a risk factor for both SIM and spontaneous myopathy [10,11]. It's important to note that hypothyroidism can sometimes manifest with minimal or even no discernible symptoms [12]. Our patient, despite having a thyroid-stimulating hormone level exceeding 100 mU/L, notably did not exhibit the hallmark signs typically associated with hypothyroidism. 

The use of statins in individuals with hypothyroidism presents significant risks [4]. While the precise biochemical mechanisms underlying hypothyroid myopathy and SIM remain elusive, it's clear that hypothyroidism amplifies the susceptibility to SIM [10-12]. Hypotheses about the origins of hypothyroid myopathy involves potential defects in glycogenolysis and impaired mitochondrial oxidation, while SIM theories include a reduction in small guanosine 5’-triphosphate-binding proteins and decreased cholesterol synthesis, which destabilizes skeletal myocyte membranes [13]. These mechanisms likely act synergistically when statins are prescribed to hypothyroid patients, with a greater likelihood of myopathy at higher statin doses. It's also worth noting that other lipid-lowering medications, such as fibrates and even ezetimibe, can induce myopathy [11,12].

While it's ideal for all patients to undergo an assessment for hypothyroidism and receive treatment to achieve a euthyroid state before initiating statin therapy, some patients do not receive such evaluations. Therefore, for individuals exhibiting suboptimal biochemical responses to lipid-lowering therapy or unexpected SIM, a retrospective review of their medical history should include an examination of prior thyroid assessments. Additionally, hypothyroidism should be considered as a potential contributing factor.

Corticosteroids are often the initial treatment choice aimed at suppressing muscle inflammation and restoring strength, frequently leading to rapid improvements in conditions like dermatomyositis, polymyositis, necrotizing myopathy, and juvenile myositis [14,15]. However, not all patients respond positively, and many may experience undesirable side effects, particularly when high doses or prolonged treatment are involved [16]. In certain cases, suspicion should arise regarding corticosteroid-induced myopathy, especially when persistent weakness affects the proximal muscles despite normal muscle enzyme activity [17]. This form of myopathy typically ameliorates with a reduction in corticosteroid dosage combined with physical exercise [16]. As there are no established guidelines, the medical approach must be tailored individually, considering factors such as the severity of clinical presentation, disease duration, the presence of extramuscular features, prior therapy, and contraindications to specific agents [17].

## Conclusions

IM presents a complex diagnostic challenge due to its multifaceted nature and association with various potential causes. A growing concern is the use of statins for hyperlipidemia, which has emerged as a significant cause of muscle-related adverse events in myositis patients. While statins are crucial for cardiovascular risk management, they pose a risk of SIM, especially in the context of coexisting hypothyroidism, a known myopathy risk factor. Our emphasis lies in personalized patient management, involving thorough biochemical monitoring of thyroid function both before and during statin therapy. Achieving a euthyroid state before initiating statins is advisable, and vigilant clinical follow-up is essential to detect early signs of SIM or myopathic symptoms. We highlight the intricate balance between the benefits and potential risks of statin therapy, particularly in the presence of conditions like hypothyroidism, underscoring the importance of meticulous monitoring and patient education for optimal treatment outcomes.
